# Strong bat predation and weak environmental constraints predict longer moth tails

**DOI:** 10.1098/rspb.2024.2824

**Published:** 2025-05-07

**Authors:** Juliette J. Rubin, Caitlin J. Campbell, Ana Paula S. Carvalho, Ryan A. St Laurent, Gina I. Crespo, Taylor L. Pierson, Robert Guralnick, Akito Y. Kawahara

**Affiliations:** ^1^ McGuire Center for Lepidoptera and Biodiversity, Florida Museum of Natural History, Gainesville, FL, USA; ^2^ Department of Biology, University of Florida, Gainesville, FL, USA; ^3^ Smithsonian Tropical Research Institute, Balboa, Panama; ^4^ Bat Conservation International, Austin, TX, USA; ^5^ Department of Entomology, Smithsonian National Museum of Natural History, Washington, DC, USA; ^6^ Museum of Natural History, University of Colorado, Boulder, CO, USA; ^7^ Florida Museum of Natural History, University of Florida, Gainesville, FL, USA

**Keywords:** biogeography, elaborate traits, insectivorous bats, predator–prey, Saturniidae

## Abstract

Elaborate traits evolve via intense selective pressure, overpowering ecological constraints. Hindwing tails that thwart bat attack have repeatedly originated in moon moths (Saturniidae), with longer tails having greater anti-predator effect. Here, we take a macroevolutionary approach to evaluate the evolutionary balance between predation pressure and possible limiting environmental factors on tail elongation. To trace the evolution of tail length across time and space, we inferred a time-calibrated phylogeny of the entirely tailed moth group (*Actias + Argema*) and performed ancestral state reconstruction and biogeographical analyses. We generated metrics of predation via estimates of bat abundance from nearly 200 custom-built species distribution models and environmental metrics via estimates of bioclimatic variables associated with individual moth observations. To access community science data, we developed a novel method for measuring wing lengths from un-scaled photos. Integrating these data into phylogenetically informed mixed models, we find a positive association between bat predation pressure and moth tail length and body size, and a negative association between environmental factors and these morphological traits. Regions with more insectivorous bats and more consistent temperatures tend to host longer-tailed moths. Our study provides insight into tradeoffs between biotic selective pressures and abiotic constraints that shape elaborate traits across the tree of life.

## 1. Introduction

Elaborate traits (complex, conspicuous derivations of pre-existing traits that serve a novel function [1]) provide a lens through which we can investigate opposing evolutionary pressures, as they are most likely to have emerged via strong selection. From the narwhal’s tusk [2] to the peacock’s train [3] to the porcupine’s quills [4], elaborate traits often play a role in high-stakes inter or intraspecific interactions—either to win potential mates or to evade potential predators. Due to their complexity and conspicuousness, these traits are commonly assumed to come with tradeoffs [5]. In some cases, tradeoffs have been empirically shown [6] but in many systems they can be hard to measure [7,8]. Frequently, when attempting to uncover tradeoffs, tests focus on short-term ‘acute tradeoffs’ (i.e. increased energy expenditure, reduced manoeuvrability, etc. [5]). It can also be difficult to estimate these acute costs, given that traits evolve as integrated components of an animal’s biology and thus commonly occur in tandem with cost-reducing characteristics [9]. As a result, longer-term tradeoffs are usually the more relevant constraining force on trait elaboration [5]. Here, we use macroevolutionary analyses to investigate the relative roles of biotic and abiotic factors on the evolution of an elaborate wing trait in moths.

Moths in the family Saturniidae typically live for only a week as adults, during which time they do not feed and must locate mates at night to reproduce [10] while avoiding echolocating bats. At least five saturniid lineages have independently evolved hindwing tails with twisted and cupped ends [11]. Live bat–moth battles have revealed that these tails function as an anti-bat strategy. Experimental alteration, as well as natural variation of tail length in the luna moth (*Actias luna*) and the African moon moth (*Argema mimosae*), showed that as tail length increases, moth escape success also increases [11,12]. Compared with individuals whose tails were removed, those with tails got away >25% more from bat attack, despite there being no measurable difference in moth flight kinematics between treatments [12]. Tails therefore represent a powerful countermeasure to a nearly ubiquitous nocturnal selective force [13] and their success is highlighted by their repeated convergence across the saturniid family tree [12].

Studies on alternative pressures of hindwing tails have thus far been unable to uncover another driver or acute tradeoff. Mating trials using the luna moth have found no evidence that tails are used in mate selection [1]. Experimental studies with tailed and non-tailed luna moth models and diurnally foraging birds indicated that tails do not increase roosting moth conspicuousness to these predators, nor do they protect the moth by breaking search image [14]. These wing appendages also do not seem to be either a hindrance nor an asset to evasive flight manoeuvres based on in-battle kinematic analysis [12].

Tails may instead be limited by longer-term tradeoffs. In general, Lepidoptera wings grow proportionally with body size and both attributes are influenced by nutrition [15,16]. The longer amount of time a lepidopteran can stay in its larval form acquiring resources, the larger its body and traits are likely to be. Developmental studies testing tradeoffs between appendages in larval and pupal butterflies also indicate that growing and shaping wings has resource allocation costs [17,18]. An evo-devo study with the sphingid moth (the sister family to saturniids [19,20]) *Manduca sexta* showed that an increase in body size comes with a compensatory increase in development time or growth rate for wings to achieve appropriate allometric scaling [21]. Thus, seasonality is expected to lead to a broad pattern where adult lepidopteran body size and associated traits are smaller in more seasonally variable environments (generally higher latitude regions) and larger in more consistent (lower latitude) environments with longer growing seasons [22–26]. Insects therefore do not seem to conform to the same ecogeographic laws that has been ascribed to endotherms. That is, body size does not necessarily increase at higher latitudes (Bergmann’s law) [24,27,28], nor do appendages (wings) appear to shorten at higher latitudes (Allen’s law) [29]. Instead, body size and wing lengths are probably governed by other physiological forces. In the case of elaborate wing structures, it may be that the energetics of building extra wing material for a tail is a limiting factor for moths living in more seasonally variable environments with shorter growing seasons.

To test the macroevolutionary pressures that have shaped the elaborate hindwing tail trait, we focused our analyses to an entirely tailed clade of Saturniidae: *Actias + Argema*. This group is mostly distributed across Asia, from present-day Russia to Indonesia, and Africa [30], thus covering a broad range of habitat with many environmental conditions and exhibiting an array of hindwing tail lengths. We hypothesized that across their distribution and evolutionary history, large insectivorous bats have exerted a positive selective force on saturniid hindwing tails, but that elongation has been constrained by abiotic environmental factors. We further hypothesized that the association between bat predation pressure and moth body size has not been as strong as the association between bats and hindwing tail length, but that body size has been similarly susceptible to environmental constraints. This biologically informed macroevolutionary approach provides a useful framework for scientists to examine the environmental and biological pressures driving trait elaboration across diverse taxa.

## 2. Material and methods

### (a) Taxon sampling and DNA extraction

To reconstruct a well-sampled phylogeny of *Actias*, we used a combination of previously published data (seven ingroup species) [12] and newly sampled specimens from the McGuire Center for Lepidoptera and Biodiversity (MGCL) at the Florida Museum of Natural History, Gainesville, FL, USA (14 ingroup species; see Dataset S1 on Dryad [31] for more details). We selected outgroup species for their use as secondary calibration points in our phylogeny from sequences published in Kawahara *et al*. [20], as this analysis is the most comprehensive, fossil-calibrated phylogeny of Lepidoptera to date. Newly sampled specimen sequences were sent to RAPiD Genomics (Gainesville, FL, USA) for library preparation, hybridization enrichment and sequencing using an Illumina HiSeq 2500 (PE100) (see electronic supplementary material, Methods for more information).

We analysed our dataset via the anchored hybrid enrichment (AHE) pipeline of Breinholt *et al*. [32], using the Bom1 probe kit (895 total loci) with *Bombyx mori* as our reference taxon [19]. We focused our analyses to coding regions (exons) and used MAFFT to align our sequences. We removed all loci that had <50% taxon coverage, leading to a total dataset of 535 nuclear loci (40% of possible loci). To ensure that each locus was in the correct frame and did not contain any spurious nucleotides, we visualized each file in AliView [33] and made any necessary manual edits. To assemble our supermatrices, we used FASconCAT-G v1.02 [34]. Cleaned probe regions and supermatrices can be found on Dryad, Archive 3 [31].

### (b) Phylogeny and estimation of divergence times

We reconstructed phylogenies with maximum likelihood (ML) and Bayesian inference (BI) optimality criteria, in IQ-TREE v. 2.0.3 [35] and BEAST v. 1.10.4 [36], respectively (electronic supplementary material, figures S1–S3, and Methods for more details on IQ-TREE methods). We also performed a multispecies coalescent analysis with ASTRAL-III (v. 5.7.5) [37], using all the default settings, and assessed branch support using local posterior probabilities where anything <0.95 is considered weak support. This tree did not conflict significantly with our ML tree and we focus our analyses to the ML and Bayesian trees.

To infer our BEAST trees, we used BEAUTI v.1.8.4 [36] to create our command file. To infer divergence times, we used four secondary calibration points from Kawahara *et al*. [20]: Lasiocampoidea/Bombycoidea + other leps (78.61–99.27 Ma), Lasiocampoidea + Bombycoidea (74.15–94.4 Ma), Sphinigidae + Saturniidae (56.86–75.42 Ma), Saturniidae (33.82–51.24 Ma), Saturniini (14.54–30.63 Ma). We built a series of Bayesian trees that varied by operator mix and tree priors for 200 million generations each, sampling every 20 000, and compared their fit with our data using ‘path sampling/stepping-stone sampling’ marginal likelihood estimates (MLE) (electronic supplementary material, table S1) [38]. All analyses were performed on the University of Florida’s high-performance computing cluster, HiperGator2.

### (c) Ancestral range estimation

**Figure 1.**
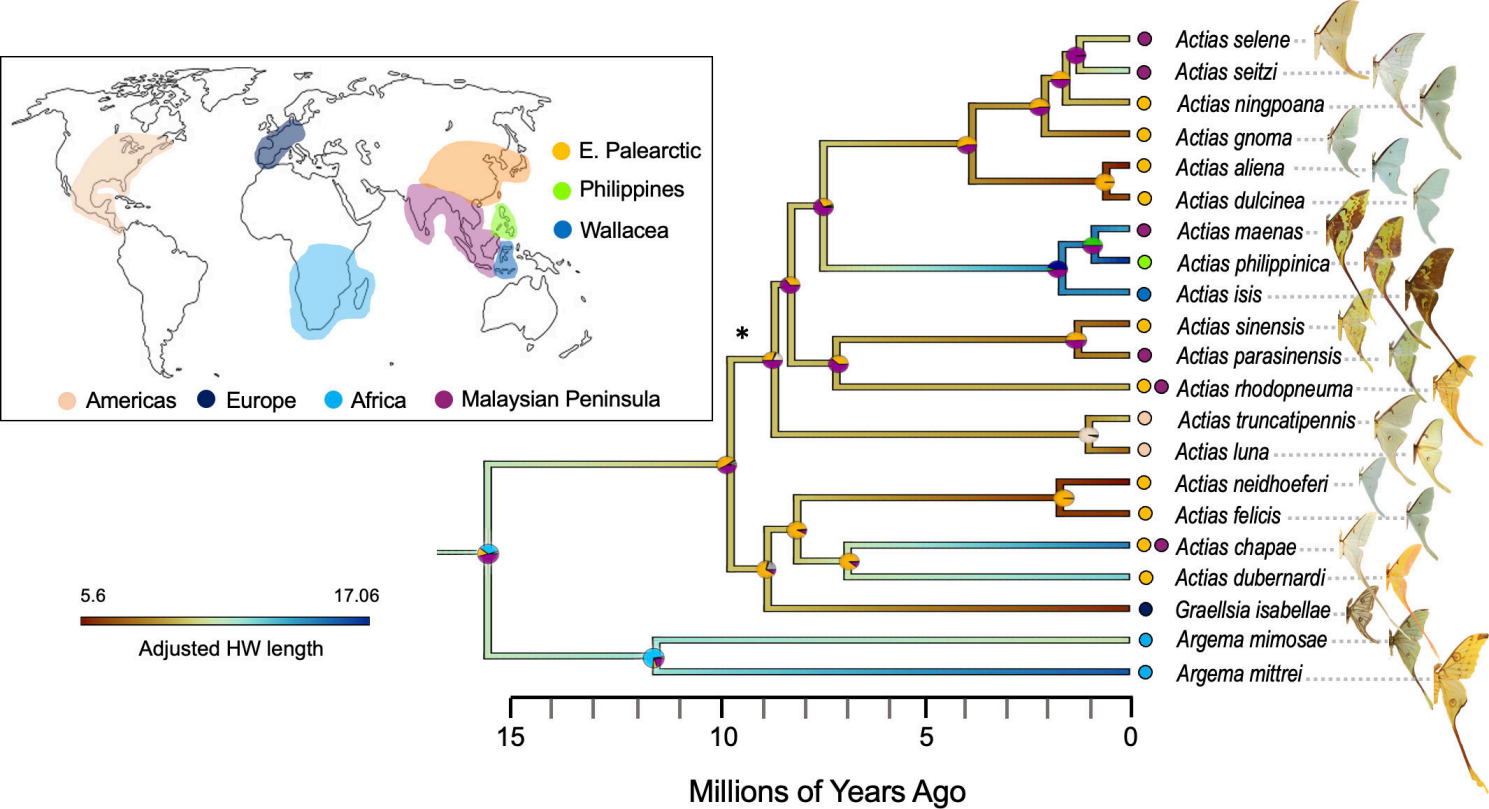
A time-calibrated tree of tailed moon moths (*Actias* + *Argema*) showing the inferred evolutionary and biogeographic history of long tails. Branches are coloured by adjusted hindwing length (hindwing length ÷ (antenna length ÷ mean species antenna length)) from images with a scale bar, with bluer colours representing longer hindwing tails and redder colours representing shorter tails. Median ages (in millions of years) were derived from a BEAST tree built with a birth–death prior using nodal calibrations from Kawahara *et al*. [20]. All support values from the starting maximum likelihood tree were 100/100, except at the node indicated by the asterisk, which was 80/100 (UFBoot/SH-aLRT). Coloured circles represent probabilities of inferred ancestral ranges from our biogeographical (BioGeoBEARS) analysis, with colours reflecting the coloured regions of the map at left.

To estimate ancestral ranges, we used the R package BioGeoBEARS [39] in RStudio (v. 2022.12.0+353) to fit a dispersal–extinction–cladogenesis (DEC) model [40]. We used our BEAST maximum clade credibility tree and pruned outgroups to focus solely on species of interest and a few most closely related sister taxa. Our information about extant distributions of tailed moon moth species came from GBIF, iNaturalist (Research Grade only), and museum collection locality data, as well as expert input (Stefan Naumann and R.A.S.L.; see Archive 4 on Dryad [31]). Following Toussaint & Balke [41] and Lohman *et al*. [42], we defined seven regions based on biogeographical patterns and barriers (e.g. oceans, mountains): Africa (F), Americas (A), Europe (E), Philippines (H), Indomalaya + Greater Sunda Islands (M), East Palaearctic (P) and Wallacea (W) (figure 1; electronic supplementary material, figure S5; geography file and dispersal multipliers can be found in electronic supplementary material, tables S2–S3 and in Archive 4 on Dryad [31]). We conducted two separate analyses, one more permissive and one more restrictive. Our permissive analysis allowed a maximum of four possible range outcomes, with nonadjacent ranges disallowed. To limit the number of permutations, and given that extant species exist in a maximum of two of our defined regions, our second analysis restricted possible range outcomes to 2 and defined the combination of regions that were possible (i.e. only adjacent regions).

### (d) Bat predation pressure

We generated species distribution models (SDMs) for bats carefully selected to represent likely saturniid predators. Our selection process identified bats that are primarily insectivorous and of sufficient size to be common predators that would exert strong evolutionary pressure on these moths, avoiding those that might occasionally pursue insect prey under limited circumstances (e.g. frugivores that may opportunistically prey upon insects, such as Phyllostomids [43]). We first identified all bat families where ≥50% of genera are aerial insectivores (18 out of 20 families) [44]. From these families we selected genera where ≥50% of species are sufficiently large (≥10 g on average) aerial insectivores whose ranges overlap our moth species of interest (30 out of 129 genera), and finally filtered the data set to just species that also followed this description (Dataset S2 on Dryad [31]). We chose this size threshold based on observations of bat behaviour in the lab [11,12] and the general scaling of bat size to prey size [45]. After filtering, 179 species (59% of our initial target list) had sufficient occurrence records to reliably fit SDMs.

We leveraged an SDM-generation pipeline optimized for generating the distribution models of hundreds of species [46,47], customized to enhance performance for bats (see electronic supplementary material, Methods for more details). We selected model predictors for SDMs based on other macroecological studies of bats [48–51]; for example, we used topological ruggedness and roughness as proxies for cave and carst roosting habitats used by bat species [48]. Initial models were fitted using 15 candidate predictors from BioClim (BIO1−2, 4−6, 12−17 [52]), three topographic (elevation, roughness and terrain ruggedness index [53]), and one from MODIS data (percentage tree cover [54]). We selected these initial candidate predictors to reduce collinearity while representing biologically plausible factors related to bat ecology. To minimize model complexity and increase reliability, we performed a series of model and manual checks (see electronic supplementary material, Methods for more details and table S4 for final models).

**Figure 2.**
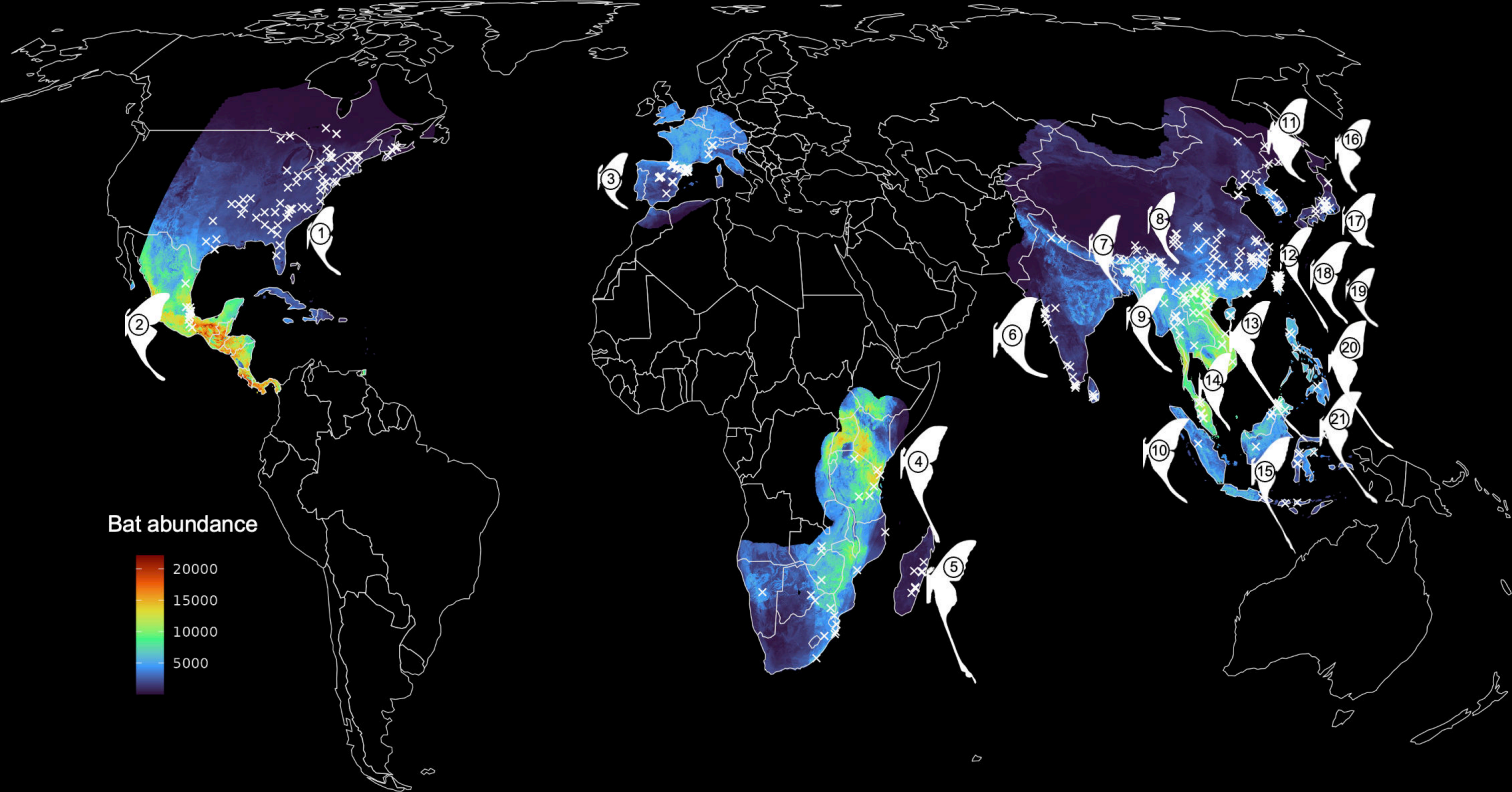
World map, pseudo-coloured by bat predation pressure (sufficiently large, insectivorous bat abundance). We limited the visualization of our spatially explicit bat abundance estimates to only those areas that overlap with moth species of interest. Moth half-silhouettes indicate the general region where the species occurs. White x marks indicate precise moth observation locations, taken from museum collections, GBIF and iNaturalist. We extracted environmental variable values from WorldClim and length of growing season values from the UN FAO FGGD LGP map for each moth point. We used these parameters, as well as measurements from the associated moth photo and estimates of bat abundance at each point, to build our phylogenetically informed models. Species names: (1) *Actias luna*, (2) *A. truncatipennis*, (3) *A*. [*Graellsia*] *isabellae*, (4) *Argema mimosae*, (5) *Argema mittrei*, (6) *Actias selene*, (7) *A. parasinensis*, (8) *A. felicis*, (9) *A. rhodopneuma*, (10) *A. seitzi*, (11) *A. dulcinea*, (12) *A. dubernardi*, (13) *A. chapae*, (14) *A. sinensis*, (15) *A. maenas*, (16) *A. gnoma*, (17) *A. aliena*, (18) *A. ningpoana*, (19) *A. neidhoeferi*, (20) *A. philippinica*, (21) *A. isis*.

We estimated species richness as the summed clog-log probability values from continuous surfaces, as recommended in [55]. To generate our estimates of bat abundance, we multiplied the clog-log probability of the SDM surface for each species by the population estimates provided in [56] and then divided by the sum of the clog-log surface to estimate the number of individual bats of a species in each 4664 m × 4664 m grid cell (see electronic supplementary material, Methods for more details). We then summed each species-specific density surface to estimate total bat density in areas that overlap the moth species of interest (figure 2). For both bat richness and abundance, we extracted values at each site associated with moth hindwing length measurements. Code used to generate bat SDMs can be found at Zenodo [57].

### (e) Hindwing tail trait acquisition

To extract wing measurements and associate these measurements with the individual’s coordinates, we gathered photographs from both natural history collections, including MGCL, American Museum of Natural History (AMNH), New York, NY, USA, and Stefan Naumann’s collection, (Berlin, Germany), and publicly sourced data repositories, including GBIF and iNaturalist (see Dryad for all photos [31]). To scrape images and associated coordinates from these online repositories, we used the function *occ_cite* in the R package ‘rgbif’ [58] (see script on Dryad, Archive 3 [31]). While iNaturalist vastly increased our number of observations per species over museum specimens alone (142 photos from museums, 327 photos from iNaturalist), photos on this site are unstandardized and most often are not associated with a scale bar. We therefore sought to find an alternative means of extracting a measurement of wing length. We determined that the most commonly visible components of the moth in these pictures were the forewings, hindwings, top-half of the thorax and antennae. This allowed us to sex the moths, as males have more highly pectinate antennae than females. We only used males in our dataset because males are better represented in both museum collections and community science photos and because they are probably subject to higher bat predation as they fly around seeking mates [59]. In bat–moth interactions, the distance between the moth’s body and tail tips are most predictive of escape success [12]. We therefore were most interested in absolute tail length for our analyses. As a result, our goal was to find a component of the moth’s body that was as agnostic to body size as possible, to be used as a relatively standardized scale bar. We found that antennae length, unlike thorax width, had a low correlation with FW length (body size) in these species (electronic supplementary material, figure S6). We verified these results with a series of Wilcoxon ranked sums tests for each species to ensure that their adjusted wing length measurements were not significantly different from their absolute (calibrated, un-adjusted) wing length measurements (see electronic supplementary material, Methods, figures S7–S8 and table S5). To ensure that our results for different species were not biased by the number of scaled or unscaled photos that were available for each, we used the adjusted wing length measurements for all of our analyses. All measurements were extracted using imageJ v.1.53t [60]. See electronic supplementary material, Methods for details on measurement and photo details.

### (f) Phylogenetically informed trait analysis and ancestral state reconstruction

To determine the strength of biotic and abiotic pressures on relative hindwing length, we used the function *pglmm* from the R package ‘phyr’, a mixed model approach to estimate evolutionary phenomena, accounting for phylogeny and spatial correlation [61]. We used adjusted hindwing length (hindwing length ÷ (antenna length ÷ mean species antenna length)) as our response variable, regardless of whether the photo had a scale bar or not. To create our abiotic predictor variables, we extracted bioclimatic and growing period data for each moth occurrence (each lat/long) in our dataset. Climatic variables came from the historical WorldClim dataset (2.5 arc-minute resolution, via the R package ‘raster’), which averages values between the years 1970−2000 (https://www.worldclim.org/data/bioclim.html), and were as follows: mean annual temperature, seasonality, and average annual precipitation. Median length of growing period (LGP) was extracted using ArcGIS Pro v.2.6.6 from the Food and Agriculture Organization Food Insecurity, Poverty and Environment Global GIS Database [62] (see electronic supplementary material, Methods for more information on these variables). The phylogenetic covariation matrix and moth species were set as random effects in our models to account for relationships between the species and multiple occurrences per species. All variables were mean centre scaled using the R function *scale* to make them comparable across varying units of measurement. To ensure that variables were not multicollinear (vif < 3 [63]), we used the *vif* function from the R package ‘car’. We ran a series of models with single or multiple predictors and used their DIC scores from the pglmm regression to determine best fit (electronic supplementary material, table S5 and Archive 1 on Dryad [31]).

We conducted comparative trait analyses and ancestral state reconstruction using the *ContMap* function and estimated phylogenetic signal using the *phylosig* function in the R ‘Phytools’ package [64]. While we used only scaled museum specimens for this analysis, we used the same adjusted hindwing metric that we used for ecological models to maintain consistency. To determine whether the best-fitting evolutionary parameter underlying this trait evolution was Brownian motion (BM; random walk), Ornstein-Uhlenbeck (OU; adaptive peaks) or early burst (EB; rapid then slow morphological evolution), we used the R ‘Rphylopars’ package [65]. We used SURFACE [66] in R to test for convergent trait regimes across the phylogeny (see code and outputs on Dryad [31]).

## 3. Results

### (a) Phylogeny and estimation of divergence times

We built a 21-ingroup species AHE tree, including 14 newly sequenced specimens and seven previously sequenced specimens (see Dataset S2 on Dryad [31] for source and preservation type for each species). This represents about half of the total species in this group (40 species [67]), however, we accomplished broad sampling across the genus and the majority of missing species are in species complexes with those that we have represented in this tree. As with other phylogenetic studies of Saturniidae (e.g. 10, 11), we recovered a well-supported monophyletic group comprising *Argema* (*Actias + Graellsia*), sister to the Australian/Papua New Guinea clade *Syntherata* (*Opodiphthera + Neodiphthera*). Based on our log marginal likelihood comparisons (electronic supplementary material, table S1), we decided to use the Bayesian fixed tree with a birth–death model for all further analyses and interpretation (electronic supplementary material, figure S3). We found that the *Actias* + allies diverged from these sister taxa approximately 21.5 Ma (HPD: 17.01–26.34 Ma) (figure 1). While *Graellsia* has been known to be nested within *Actias* [68], and this was confirmed in our study (divergence from the other *Actias* in its clade approx. 8.5 Ma, HPD: 5.99–10.59 Ma), we maintained the convention of using the *Graellsia isabellae* nomenclature. Subsequent to completing our analyses and this paper being provisionally accepted, an article was published definitively renaming *Graellsia isabellae* as *Actias isabellae* [69]. We agree with this name change, but have kept the original name under which we completed all our analyses. Within the *Actias +* allies ingroup, our tree largely agreed with the typology of previous, less densely sampled AHE trees, as well as a study that reconstructed a phylogeny based on 16 *Actias* + allies species based on molecules, morphology and behaviour [70].

We found that Brownian motion was the best fitting evolutionary model underlying the hindwing trait. That is, the evolution of tail length can be best described by a random walk, in comparison with a model with adaptive peaks or an early burst model (see scripts in Archive 1 on Dryad [31]). In line with this result, we found significant phylogenetic signal in hindwing length (where greater phylogenetic signal is represented by a *K* value closer to 1; Blomberg’s *K* = 0.78, *p* = 0.002), and our SURFACE analysis [66] revealed only one hindwing length regime shift at the stem of *Argema + Actias* from the non-tailed sister taxa (electronic supplementary material, figure S9). While we did not detect a signal of adaptive peaks in our dataset, our ancestral state reconstruction (ASR) analyses indicate that tails have repeatedly elongated in at least three separate lineages: *Argema mittrei + A. mimosae, Actias chapae + A. dubernardi* and *Actias maenas + A. philippinica + A. isis*. We also find evidence of tail length shortening in an equal number of lineages: *Graellsia isabellae, Actias neidhoeferi + A. felicis* and *A. aliena + A. dulcinea* (figure 1). For comparative trait analyses using absolute (calibrated, unadjusted) hindwing length and forewing length, see electronic supplementary material, figures S10–S11.

### (b) Ancestral range estimation

To examine whether biogeographical history could explain some of the variation in hindwing tail length, we used BioGeoBEARS [39] to estimate ancestral ranges. Our 4-range limit analysis resulted in unlikely combinations of ranges, however (electronic supplementary material, figure S5a), and we therefore interpret results from the 2-range limit analysis (figure 1; electronic supplementary material, S5b). Based on the inferred ancestral range of the common ancestor between *Actias* + allies and their closest sister genera, we think it likely that this group was present in the Indomalaya region by at least 21.5 Ma (0.91 probability that Indomalaya is in the ancestral range). We inferred a 0.83 probability that Indomalaya is part of the ancestral range of the tailed moon moths (*Argema* and *Actias* species) and a 0.62 probability that Africa is in the ancestral range. The lineage leading to *Argema* split off from the rest of *Actias* and made it to Africa by about 11 Ma (HPD: 4.98 - 14.98 Ma; 0.97 probability). *Actias* then diverged into a Palaearctic group and an Indomalaya group by approximately 9.5 Ma (HPD: 6.07–11.45 Ma). It appears that there was a second wave of *Actias* movement into the Palaearctic region by about 3.5 Ma (HPD: 2.4–5.04 Ma), leading to the extant short-tail species *A. dulcinea, A. aliena* and *A. gnoma*. The diversification of *Actias* species across Wallacea and the Philippines islands approximately 1.5 Ma (HPD: 0.69–2.18 Ma) probably originated from populations in Malaysia (0.91 probability). It is unclear how *Actias* arrived in the Philippines, as there is a roughly equal likelihood that they colonized this region via Wallacea or from the Indomalaya region. We also do not have strong inference as to the exact manner in which *Actias* colonized Northern America and Europe, but our analysis indicates that they did so from the Eastern Palaearctic region, with lineages leading to *A. luna* and *A. truncatipennis* probably using the Bering Land Bridge (figure 1; electronic supplementary material, figure S5).

### (c) Phylogenetically informed linear mixed models

**Figure 3.**
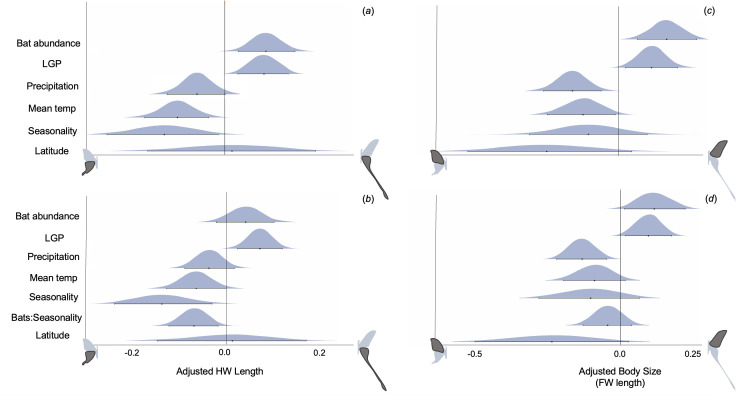
Tail length and body size in the tailed moon moth group are positively related to bat predation pressure and constrained by environmental factors. We find that insectivorous bat abundance is positively correlated with (*a*) hindwing tail length and (*c*) body size (forewing length) in *Actias* + *Argema* (Saturniidae). Length of growing period (LGP) is also positively correlated. Average annual precipitation, average annual temperature and seasonality (standard deviation of temperature across the year) are negatively correlated with both tail length and body size. Thus, areas with higher bat abundance and longer periods of plant productivity are associated with longer tailed moth species. When we include an interaction term between bat abundance and seasonal temperature variation (bat:seasonality) for (*b*) tails and (*d*) body size, we find that some of the power is removed from bats as a driver of tail length. Although this parameter overlaps the zero line, there is still an approximately 0.90 probability that bats have a positive relationship with tail length. This indicates that while bat abundance and seasonality have their own relationship with each other, they both still have independent effects on moth tails. Central tendency dots indicate parameter estimates and error bars are 95% credible intervals from the best fit phylogenetically informed linear regression analyses. All predictor variables are mean centre-scaled to make them comparable across units. Adjusted hindwing and forewing lengths are wing length/(antenna length/mean species antenna length).

Our global phylogenetically controlled linear regression model (pglmm) revealed that hindwing length exhibits a positive relationship with bat predation pressure (parameter estimate (PE) of bat abundance: 0.082, 95% credible interval (CI): 0.028–0.137; figure 3*a*). We also found a positive association between median growing season period and hindwing tail length (PE: 0.082, CI: 0.034–0.131). The credible interval for mean annual precipitation overlapped zero, but the probability that this parameter had a negative relationship with tail length was 0.94 and thus we interpret this along with the other environmental variables (PE: −0.045, CI: −0.096–0.006). Average annual temperature and annual seasonal temperature variation also displayed negative relationships with hindwing length, with seasonal temperature variation having the greatest effect size (PE seasonal temp variation: −0.127, CI: −0.236 to −0.018, PE avg temp: −0.100 CI: −0.163 to −0.039; figure 3*a*). While we felt it was important to include latitude in our models, due to its relevance in many other macroevolutionary studies, its posterior distribution was very broad, overlapped the zero line in all models, and it uniformly increased the DIC scores of our models. It did not change the relationships between our response variables and other parameters, however. We therefore maintained it in our models but do not discuss it as an important predictor (figure 3; and see code and outputs in Archive 1 on Dryad [31]).

Overall, we found that the global model performed better than a null model (which only accounts for phylogenetic relationships) and a model that contained all abiotic variables and excluded bat abundance, and performed slightly worse than an interaction model between bat abundance and seasonal temperature variation (DIC full model: 348, DIC null: 370, DIC no bats: 354, DIC interaction: 344). When we include the interaction, we find the same relationships between our parameters and hindwing tail length as in our global model. Under this framework, the bat parameter crosses the zero line, however there is still a 0.91 probability that bat abundance has a positive relationship with hindwing tail length (figure 3*b*). We found that while phylogenetic relationships alone explain much of the variance in hindwing length (*r*^2^ = 0.87), the global model explained more (*r*^2^ = 0.89). Additionally, removing bat abundance from the model decreased the explanatory power of the model by approximately 2% and including the interaction term increased explanatory power by approximately 1%, compared with the global model (*r2_pred* [71]; see code and outputs on Dryad [31]). Breaking the dataset down by moth species also demonstrated that hindwing length was positively correlated with bat abundance in almost all species and was negatively correlated with seasonal temperature variation in almost all species (electronic supplementary material, figure S12, table S6 for model structures).

The global pglmm analysis on forewing length (a proxy for body size [72,73]) showed similar relationships with all parameters (PE bat abundance: 0.146, CI: 0.062–0.233, PE LGP: 0.105, CI: 0.028–0.183, PE precipitation: −0.135, CI: −0.215 to −0.054, PE avg temp: −0.118, CI: −0.215–0.021, PE seas temp: −0.102, CI: −0.271 to −0.067) (figure 3*c*). Again, the model containing all parameters was a better fit than the null (DIC full model: 783, DIC null model: 803) and also had a better fit than the interaction model or the model without the bat abundance parameter included (DIC interaction model: 785, DIC no bats: 794). For body size, the interaction between bat abundance and seasonal temperature variation is not significant (figure 3*d*). Overall, the predictors explained less of the variance in body size (*r*^2^ = 0.72) than hindwing length (see code and outputs in Archive 1 on Dryad [31]).

## 4. Discussion

Combining species observations from iNaturalist and museum collections, a densely sampled *Actias* phylogeny (figure 1), biogeographical inference, and a comprehensive set of species distribution maps (SDMs) for 179 insectivorous bat species (figure 2), we investigated the relationship between hindwing tail length and biotic and abiotic drivers in the entirely tailed *Actias* + allies clade of Saturniidae (figure 3). Our phylogenetic and biogeographical analyses indicate that *Argema + Actias* diverged from their non-tailed sister taxa approximately 20 Ma and *Argema* and *Actias* diverged approximately 15 Ma, probably when the lineage leading to *Argema* moved to Africa and the rest of *Actias* spread from the Indomalaya region (figure 1). Subsequently, *Actias* moved throughout the Eastern Palaearctic (approx. 10 Ma) then the Western Palaearctic and Americas (approx. 9 Ma), probably beginning from an intermediate-tail length ancestor and alternately undergoing tail lengthening or shortening as species divided and moved into new areas (figure 1). Over the course of this time, the climate [74] and land masses were similar to current-day conditions, aside from movements of the Indo-Australian archipelago that continued until approximately 5 Ma (before *Actias* were present in this region, approx. 2 Ma) [42] and the closure of the Bering land bridge, which may have facilitated the movement of *Actias* to the North American continent [75,76]. The young age of this clade is therefore one of the strengths of this study, as present-day patterns can be more reliably used to infer historic dynamics. Similarly, predation pressure has probably been relatively consistent throughout the evolution of *Actias*. Based on fossil evidence [77,78] and biogeographical reconstructions [77,79–82], large insectivorous bats had already become globally spread by this time (approx. 15 Ma). Moreover, a recent comprehensive mammal phylogeny inferred an increase in speciation rates among many bat lineages approx. 10−15 Ma [83]. This rise in bat diversity and widespread prevalence of these predators could have made hindwing protrusions more profitable, as the night sky filled with more echolocators exploiting a greater depth of the prey community [84].

Analysing these macroevolutionary data in a phylogenetically informed linear mixed model framework provides evidence that bat predation pressure has probably exerted a selective force on the length of hindwing tails, while seasonal temperature variation has exerted a counterbalancing constraint on hindwing length (figure 3*a*). Moths with long tails are therefore more likely to be found in areas with more bats and fewer temperature fluctuations across the year. This result is supported by the positive association between the length of growing period and hindwing tail length. In essence, areas with longer periods of high plant productivity and more consistent temperature regimes appear to be more permissive of the evolution of long hindwing tails than areas with more restrictive seasons. Although weaker than the seasonality parameters, we found a negative association between hindwing length and average annual temperature and precipitation, indicating an opposite trend from Allen’s rule for endotherms, where appendages are expected to elongate in hotter, drier environments (as in [85]). This aligns with previous work indicating that Lepidoptera wings are not used for heat venting [86]. We did not find an effect of latitude in any of our models, signifying that the underlying drivers of wing trait evolution in this group are more complex than general latitudinal gradients. Additionally, while previous studies have found latitude to be an important correlate of bat diversity [87], others have found that it is not the most informative predictor, especially in the case of insectivores [88–90]. In congruence with this, we found relatively weak associations between insectivorous bat abundance and any of our climactic variables in the context of our global models (vif scores < 3; see code and outputs in Dryad [31]). We did find an interaction effect between bat abundance and seasonal temperature variation in relation to tail length, however, indicating that bats and seasonality have their own relationship that influences tail length. That is, areas with less seasonal variability tend to host more bats as well as longer tailed moths (and vice versa, see electronic supplementary material, figure S13 for an illustration of this interaction). From this interaction model, we also find that both seasonal temperature variation and bat abundance have their own appreciable effect on hindwing tail length. While the bat posterior distribution overlaps the zero line, there is a 0.91 probability that bat abundance is positively associated with moth tail length (figure 3*b*). Thus, the effects of predators and environment on moth hindwing tails are not confounded in this study. We note that different bat species may exert differing predatory pressures on saturniid moths based on the specifics of their echolocation strategy or feeding guild [91], but given the large-scale nature of our data set and the generally similar diets of these aerial insectivores, we have considered insectivorous bats as a pooled group for the purposes of this study. Together, our analyses indicate that tails are locked in evolutionary tension between abiotic constraints and biotic pressure.

Contrary to our predictions, body size (forewing length) demonstrated an almost identical positive association with bat abundance as hindwing length (figure 3*c*,*d*). This could be because wing/body sizes are tightly integrated such that long hindwing tails require, or are made possible by, larger body sizes. Rather than simply being a necessary precursor for long hindwing tails, however, body size may be an anti-bat trait in itself. Bats seem to target prey relative to their own size, such that smaller bats eat smaller insects and larger bats are the main predators of large moths and beetles [92,93]. Whether this is due to handling, gape size or echolocation limitations is still debated [45,94–96]. We found that the positive association between bat abundance and forewing length is complemented by a positive association between length of growing period and forewing length, again indicating that longer periods of forage availability allows for longer periods of larval feeding and larger adult body sizes [15]. These effects were to some extent countered by a negative association with precipitation. This may indicate a limitation on body size in regions with more rainfall, perhaps due to hampered foraging or increased larval mortality during bouts of heavy rain [97]. However, precipitation parameters from WorldClim should be considered with caution, especially from tropical regions with fewer climactic field data collection stations [98].

In addition to its use in our statistical models, comparative trait analyses revealed multiple origins of tail elongation but only one adaptive peak at the stem of the long-tailed moon moth clade, comprising all tailed species. This may be a result of the relatively limited number of species in this group and the strong phylogenetic signal underlying the tail trait. That is, while hindwing length varies considerably among these species, all species in this clade have tails, possibly making it more difficult to find the valleys between the morphological peaks [66,99]. The multiple elongation and shrinkage events across our phylogeny indicates that the tail is a labile trait that could have become enhanced under conditions of high enough echolocating predator pressure and permissive environmental conditions, and that could relatively easily regress under more restrictive conditions. Body size, estimated by forewing length, was a far less labile trait, however (electronic supplementary material, figure S11). Previous research into the morphological lability of the fore- and hindwings of tailed swallowtail butterflies (Papillionidae), found similarly elevated hindwing shape diversity [100]. Lepidopteran wing shape variation is probably driven by different biological pressures on the two sets of wings, where forewings are essential for flight, while hindwings are helpful for manoeuvrability, but not entirely necessary [101–103]. Further, experimental evidence indicates that rather than being purely flight-driven, hindwings can play an important role in deflecting predators both during the day (in butterflies) [104] and night (in moths) [11,12].

While there are risks to making assumptions about past predator and prey dynamics based on extant forms, interactions or distributions [105], the relative consistency of environmental conditions and bat presence strengthens our inferential power. Additionally, while our bat abundance estimates come with necessary assumptions and levels of uncertainty (e.g. species distribution models can be unreliable for species that are difficult to ‘observe’, as is the case with some insectivorous bats [106,107] and the population estimates were built from a global mammal dataset which could only provide coarse estimates [56]), we are ultimately interested in relative, rather than absolute, predator abundance. In general, species richness—the backbone upon which we built our abundance estimates—remains stable when ecological limits (most driven by climactic variables) are similar [108–110]. Thus, while extant bat distributions may not directly mirror historical ones, moths were clearly under intense selection pressure by echolocating bats in these regions.

In sum, results from this study, in conjunction with previous behavioural work [11,12] provide synergistic compelling evidence that predation pressure is associated with the elongation of hindwing tails in moon moths. Considering the absence of alternative selective forces (i.e. reproduction [1] or diurnal predation [14]) and the clear efficacy of short tails to increase escape success [12], we postulate that bat predation pressure drove the origins of the hindwing tail in Saturniidae. Hindwing tails with twisted and cupped ends have emerged five independent times across Saturniidae, three times in the Saturniinae (tribes: Saturniini, Attacini, Urotini/Bunaeini), once within the Arsenurinae [11,12,72], and once in Cercophaninae [19,111]. Phylogenetic inertia and the seemingly easily modifiable unit of wing imaginal discs in developing Lepidoptera [112] probably played a role in the evolution of tails. By contrast to the adaptive anti-predator benefit of tails, we found evidence for an environmentally mediated long-term cost of these appendages. Developmental studies are needed to uncover the mechanism by which environment constrains tail enhancement. Here, our study adds an important macroevolutionary lens to previous experimental predator–prey work. Uniting these two levels of information provides important advancement to our understanding of complex evolutionary dynamics and opens new lines of inquiry for future research [113]. Additional studies at an intermediate scale, testing the relationship between microhabitat, bat predation and hindwing tails, could also reveal important detail about these dynamics. We emphasize the strength of multi-scale investigation for illuminating the relative pressures of competing eco-evolutionary forces that have shaped the origin and diversification of elaborate traits across taxonomic systems.

## Data Availability

All data and code pertaining to moth sequences, wing measurements, phylogenetic inference, biogeographical inference and phylogenetically informed linear models are available from Dryad [31]. All code pertaining to bat species distribution models (SDMs) and abundance estimates are available from Zenodo [57]. Supplementary material is available online [114].
